# Supplementary material for: Effects of Endurance and Endurance–Strength Training on Endothelial Function in Women with Obesity: A Randomized Trial
Source: Int J Environ Res Public Health. 2019 Nov 5;16(21):4291. doi: 10.3390/ijerph16214291 (PMC6862069; doi:10.3390/ijerph16214291)
Supplement: Supplementary file 1 [file ijerph-16-04291-s001.pdf]

SUPPLEMENTARY MATERIAL

Figure S1. Training volume diagram of each session for both interventions

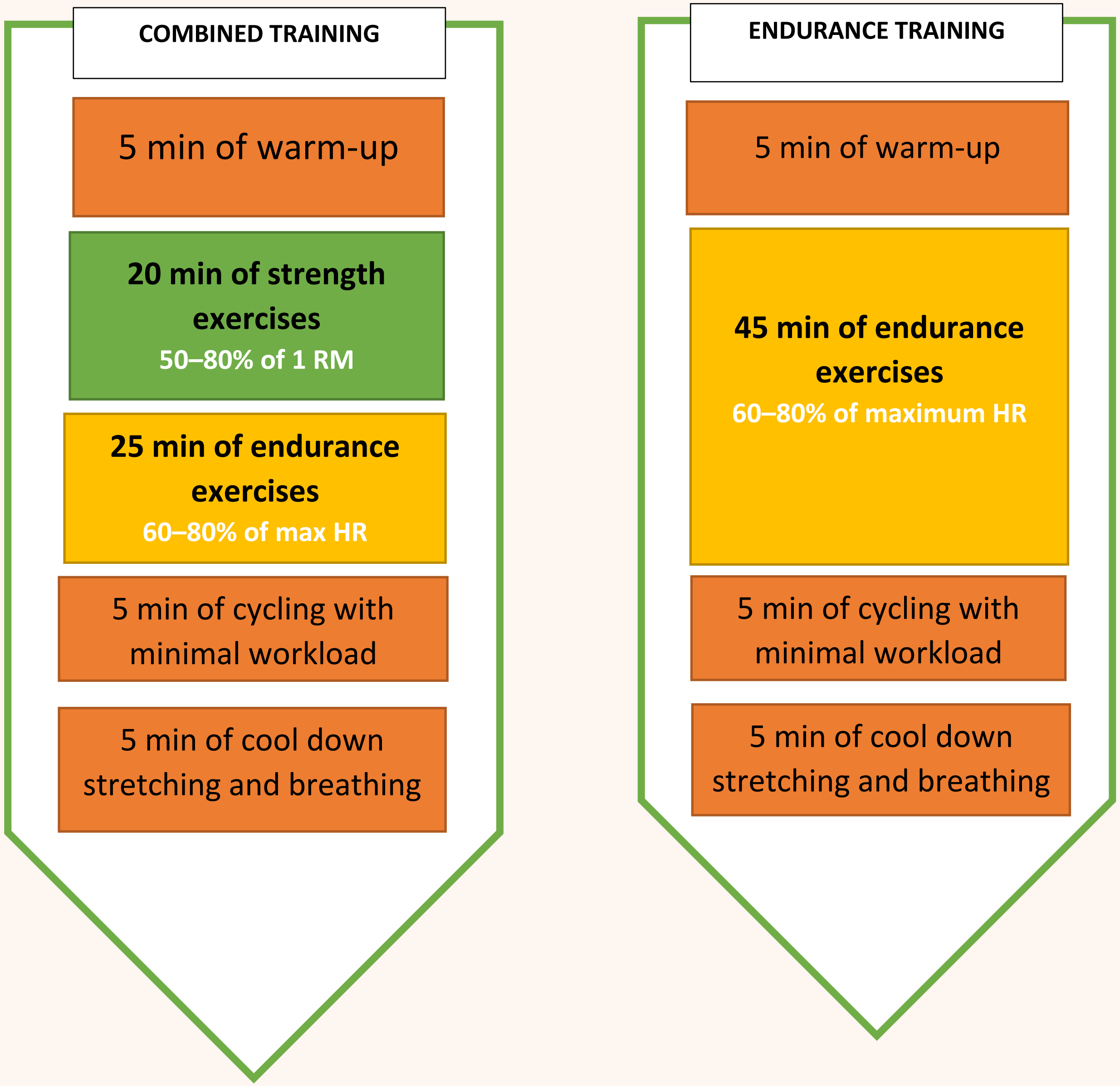

The 3-month intervention consisted of physical exercise program including 3 sessions of training per week, with a total of 36 training sessions for each group. The training programs of both groups were comparable in exercise volume, and varied only in the nature of the effort (Figure S1).

Endurance training consisted of a 5-min low-intensity warm-up (stretching exercises; 50–60% of maximum heart rate (HR), 45 min of training at an intensity of 60–80% of maximum HR, 5 min of non–weight-bearing cycling, finishing with 5 min of low-intensity warm-down stretching and breathing exercises (Figure S2).

Combined endurance–strength training consisted of a 5-min low-intensity warm-up (stretching exercises; 50–60% of maximum HR), 20 min of strength exercises using a barbell and gymnastics ball, 25 min of endurance exercise using cycle ergometers at an intensity of 60–80% of maximum HR, 5 min of non–weight-bearing cycling, and 5 min of low-intensity cool down stretching and breathing exercises (Figure S3). Due to the need for regeneration of muscle power, the strength component was variable and repeated each week. On Mondays, upper limb exercises were performed with a neck barbell; Wednesdays involved spine-stabilizing exercises, deep muscle-forming exercises, and balance-adjusting exercises with a gymnastic ball; on Fridays, lower limb exercises with a barbell were carried out (Table S1). The number of repetitions in the sets was dependent on the subjects' capabilities and was equal to the number of repetitions performed correctly ≈ 50–80% of their one-repetition maximum. The number of repetitions was systematically increased with the increase in subject's muscle strength. HR during both physical trainings was monitored with a Suunto Fitness Solution® device (Suunto, Vantaa, Finland).

EXAMPLE OF ENDURANCE TRAINING CLASS

Figure S2. Bar graph showing intensity of endurance training on cycle ergometer (Schwinn® Evolution®) based on heart rate chart (Suunto Fitness Solution®) of the selected participant

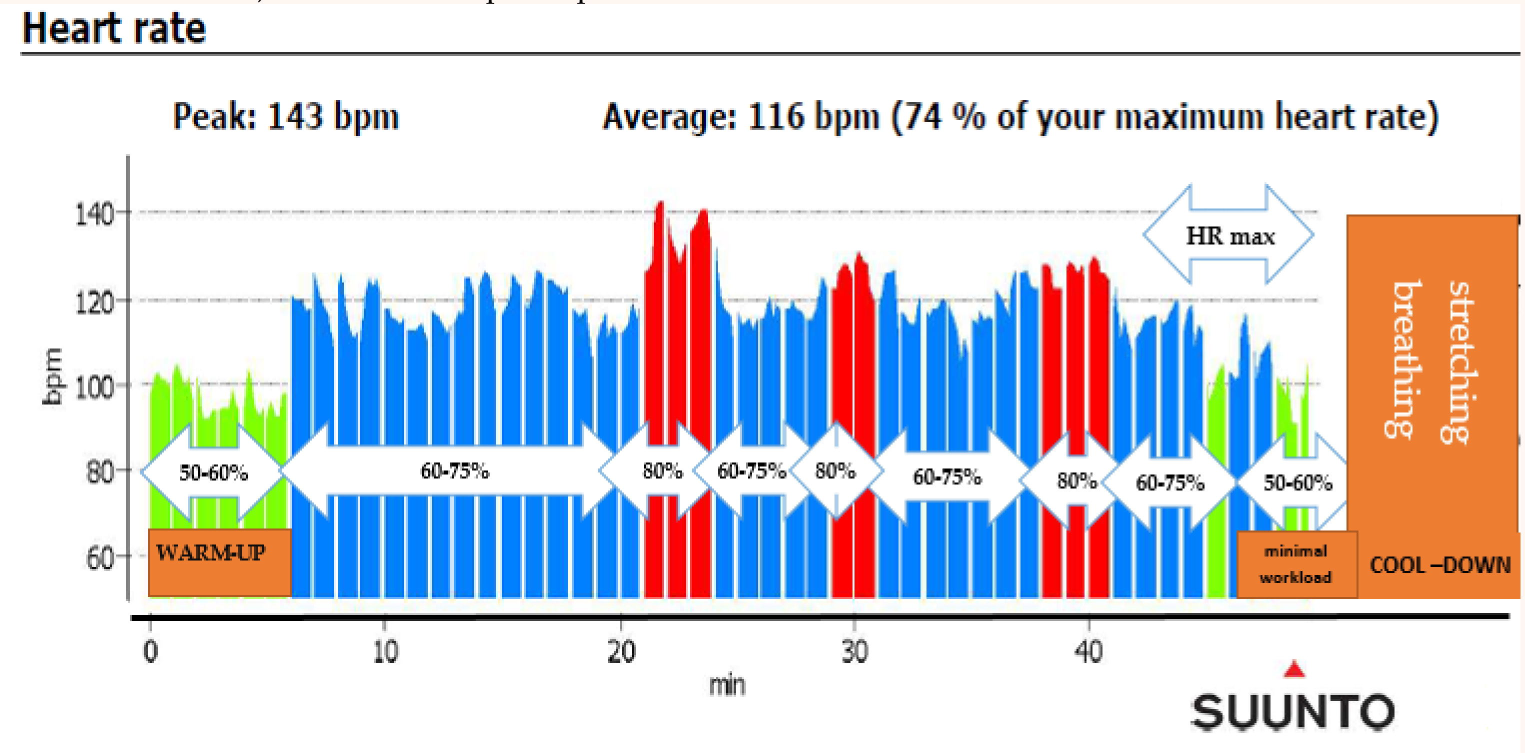

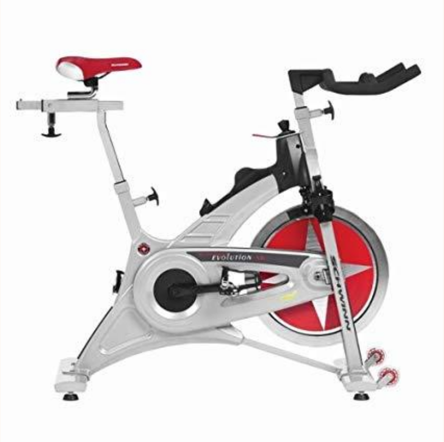

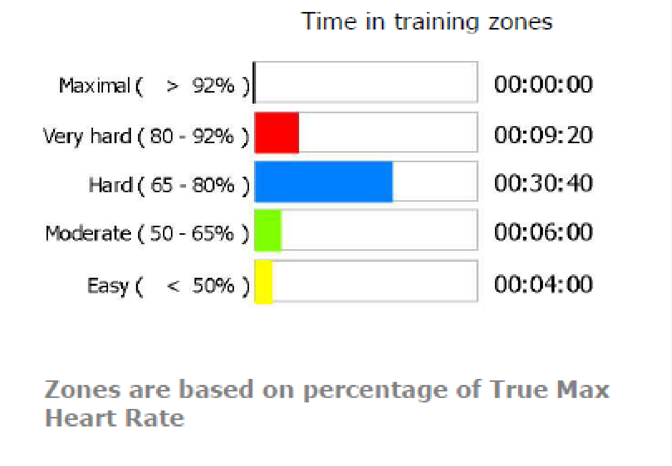

EXAMPLE OF COMBINED TRAINING CLASS

1. Warm-up – 5 minutes

2. PART I- STRENGTH EXERCISES - 20 minutes

Table S1. Structure of strength exercises over the week

**Day**	**Monday** **Upper limb exercises with a neck barbell**			**Wednesday** **Spine-stabilizing and balance exercises with a gymnastic peanut ball**			**Friday** **Lower limb exercises with a neck barbell**		
**Exercises**	**1.** Standing position. Barbell overhead press. After 3 sets- 1-minut break.	**2.** Standing position. Barbell behind neck press. After 3 sets- 1-minut break.	**3.** Standing position. Barbell curl in a full range of motion.	**1.** Kneeling straight on peanut type gymnastic ball (perpendicular to the long side of the peanut ball) - maintaining balance.	**2.** Lying forward on a peanut type gymnastic ball (lying along a peanut ball with a possibility to hold on the ball) - keeping the balance on the ball so that no part of the body touches the ground.	**3.** Straddle sit on the peanut ball (the body should not touch the ground).	**1.** Barbell squats to the right angle (barbell behind the neck) in a moderate pace.	**2.** Barbell squats to the right angle (barbell behind the neck) in a variable pace (1 x slow, 1x fast, 2x slow, 2x fast, 3x slow, 3x fast, 4x slow, 4 x fast, 5x slow, 5x fast).	**3.** Isometric contractions with knees bent to the right angle squat in a variable duration, according to the scheme: 10s, 15s, 20s, 25s, 30s, 25s, 20s, 15s, 10s.
	Between sets, participants performed a 10–15s isometric exercises with the elbows bent halfway (at 90 degree).								
**Number of sets/repetitions**	3/16 *	3/16 *	3/16 *	Depending on the level of advancement, the goal of the exercise is to maintain balance as long as possible.			6/30 *	6/30 *	9 *

*The number of repetitions in the set was dependent on the subject's muscle strength and was equal to the number of repetitions performed correctly estimated to be 50–80% of their one-repetition maximum

3. PART II- ENDURANCE EXERCISES- 25 minutes (immediately after strength exercises)

Figure S3. Bar graph showing intensity of endurance exercises in a combined training on cycle ergometer (Schwinn® Evolution®) based on heart rate chart (Suunto Fitness Solution®) of the selected participant

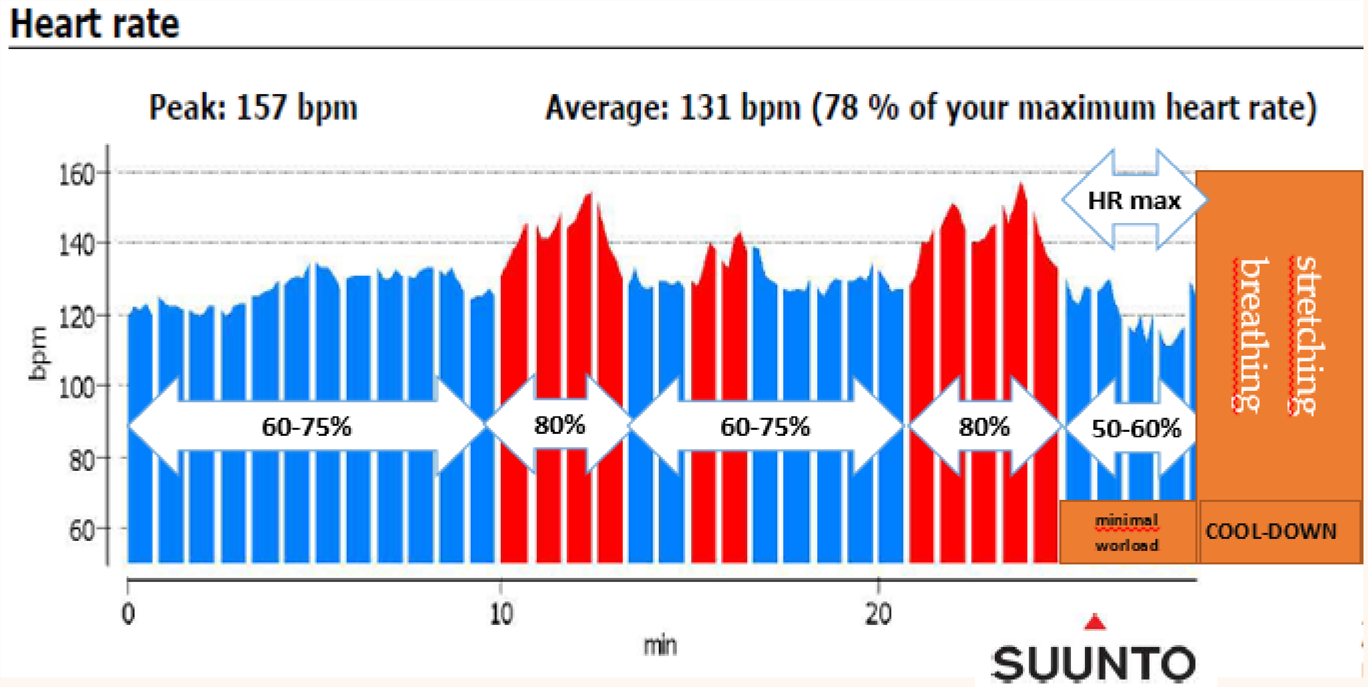

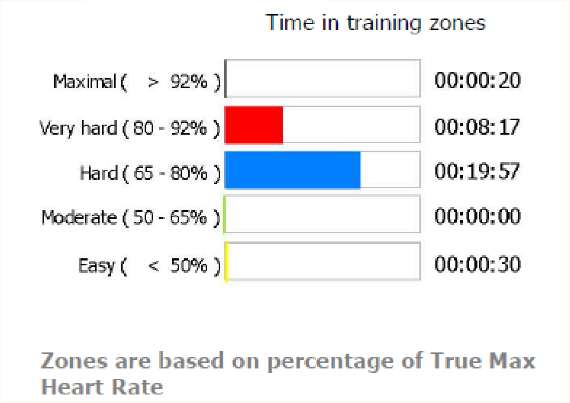

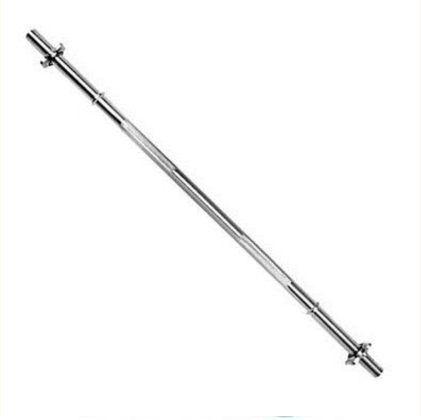

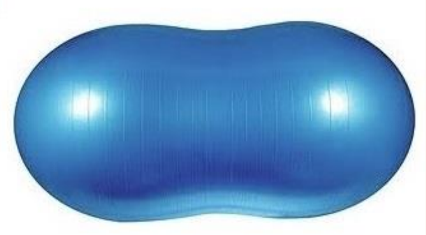

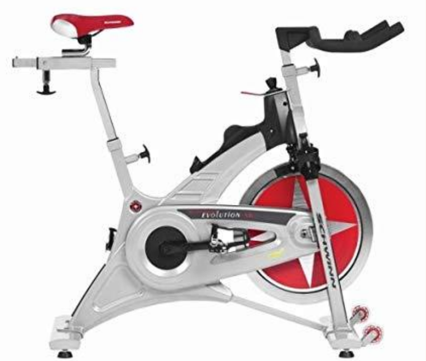

4. Cycling with minimal workload- 5 minutes

5. Cool-down, stretching and breathing exercises- 5 minutes
